# The evolving role of checkpoint inhibitors in the treatment of Hodgkin lymphoma

**DOI:** 10.3389/fonc.2024.1392653

**Published:** 2024-10-22

**Authors:** Amanda F. Cashen

**Affiliations:** Washington University School of Medicine, Siteman Cancer Center, St. Louis, MO, United States

**Keywords:** Hodgkin lymphoma, nivolumab, pembrolizumab, checkpoint inhibitors, brentuximab vedotin

## Abstract

Since their initial approval as single agent therapy for multiply relapsed/refractory Hodgkin lymphoma (HL), the PD-1 inhibitors nivolumab and pembrolizumab have been incorporated into second-line salvage regimens, and they are being investigated in upfront therapy of newly diagnosed patients. As second-line therapy in combination with brentuximab vedotin or multi-agent chemotherapy, nivolumab and pembrolizumab provide high complete remission rates and durable progression-free survival after consolidative autologous stem cell transplant. Incorporation of these agents into frontline chemotherapy regimens is feasible, and early results from a Phase III trial of nivolumab-AVD compare favorably with the existing standard for advanced stage HL, brentuximab vedotin plus AVD. As nivolumab and pembrolizumab move into earlier lines of HL therapy, open research questions include the efficacy of checkpoint inhibitor regimens in patients who relapse after frontline exposure to nivolumab or pembrolizumab; the selection of patients with relapsed HL who can achieve durable remissions without autologous stem cell transplant; and the efficacy of the PD-1 inhibitors in the frontline therapy of patients with early stage Hodgkin lymphoma.

## Introduction

Over the past five years, the treatment of newly diagnosed and relapsed/refractory Hodgkin lymphoma (HL) has rapidly evolved as novel agents are incorporated into earlier lines of therapy. The checkpoint inhibitors nivolumab and pembrolizumab have been a key component of the recent advancements in HL treatment. These antibodies against programmed death-1 (PD-1) interrupt the interaction between PD-1 on cytotoxic T-cells with PD-L1 and PD-L2 ligands on malignant cells. Hodgkin Reed-Sternberg (RS) cells reside within a tumor microenvironment comprised of a dense infiltrate of normal immune cells, but they evade the immune system via loss of major histocompatibility complex expression and overexpression of PD-1 ligands. PD-1 blockade restores T-cell effector function, allowing immune-mediated elimination of the malignant RS cell (1–3).

Initially studied and approved in third or later lines of therapy, the PD-1 inhibitors nivolumab and pembrolizumab have now been incorporated into second-line salvage regimens and most recently into front-line therapy of advanced stage HL. Phase II and III trials in these settings have demonstrated high response rates and good tolerability, and clinicians in the United States have responded with rapid adoption of PD-1-containing regimens. I will here review the evolving use of PD-1 inhibitors in the treatment of HL, with a focus on their incorporation into second line salvage regimens and the most recent data in frontline regimens.

## PD-1 inhibitors in relapsed/refractory HL

As single agents, the PD-1 inhibitors have high response rates and provide durable remissions for a subset of patients with relapsed or refractory HL. In the pivotal Phase II study of nivolumab, 243 patients with HL relapsed after autologous stem cell transplant (autoSCT) were treated with nivolumab 3 mg/kg every 2 weeks until progression or intolerance (4). Sixteen percent of patients achieved complete remission (CR) and 53% achieved partial remission (PR), with median duration of response of 22.2 months and 15.1 months, respectively. Similarly, durable responses are seen with single agent pembrolizumab, especially for those who achieve a CR. In the pivotal Phase II study of pembrolizumab, 210 patients were treated with pembrolizumab 200 mg every 3 weeks. Overall response rate was 72% (28% CR), with a median duration of response of 16.5 months (5, 6). In a Phase III trial of patients with relapsed/refractory HL, pembrolizumab showed superior response rate and progression-free survival (PFS) compared to the anti-CD30 antibody-drug conjugate brentuximab vedotin (BV) (response 66% v 54% and PFS 13.2 v 8.3 months) (7). Pembrolizumab and nivolumab have also demonstrated efficacy when given as consolidation therapy after autoSCT (8, 9). In single arm studies, nivolumab given for 6 months after autoSCT was associated with 6-month PFS of 92%, and pembrolizumab given for 8 cycles after autoSCT was associated with 18-month PFS of 82%.

Given the activity of nivolumab and pembrolizumab in patients treated with two of more prior lines of therapy, it was logical to move them to second line therapy in combination with chemotherapy. The primary goal of first salvage therapy is CR, as most patients are candidates for consolidative autoSCT, and CR before autoSCT is highly correlated with outcomes. The CR rate and post-autoSCT PFS observed with PD-1 containing regimens compare favorably both with ICE (ifosfamide/carboplatin/etoposide) and with regimens that incorporate BV (**Table 1**). Although no randomized trial has compared standard platinum-based chemotherapy to PD-1-containing regimens, a retrospective analysis of 981 patients who underwent autoSCT for relapsed/refractory HL found that PFS was significantly higher after PD-1 containing salvage regimens compared to BV- and chemotherapy-based regimens (2 year PFS 93% v. 74% and 72%) (10).

**Table 1:** Second-line salvage regimens for relapsed/refractory HL.

Regimen	N	CR %	PFS, %
ICE/GVD (36)	97	77	70 at 5 yr
BV plus:			
-bendamustine (37) -ICE (38) -DHAP (39) -gemcitabine (40)	55 45 55 42	74 74 81 67	63 at 2 yr 80 at 2 yr 74 at 2 yr not reported
BV-nivolumab (11)	91	67	77 at 3 yr
BV-nivolumab (14)	44	59	
Nivolumab +/- nivolumab-ICE (41)	42	91	72 at 2 yr
Pembrolizumab-ICE (16)	42	87	87 at 2 yr
Pembrolizumab-GVD (18)	39	95	96 at 30 mo

The combination of BV and nivolumab (BV/nivo) is notable for providing a high response rate without use of standard chemotherapy. In a multicenter Phase I-II study, 91 adult patients in first relapse were treated with up to four cycles of BV/nivo before consolidative autoSCT (11). The study enrolled a high-risk population—42% primary refractory and 30% relapsed within one year—although none had received prior BV or checkpoint inhibitors. The overall response rate was 85%, and CR rate was 67%. Seventy-four percent of the study population proceeded directly to autoSCT, and their PFS at 3 years was 91%. An additional 17 patients (17%) went to autoSCT after additional salvage chemotherapy, and their 3-year PFS was 100%. The BV/nivo regimen was well-tolerated, and only two patients discontinued treatment due to adverse events. Infusion reactions occurred in 43%, most commonly at cycle 2, and were manageable with pre-medications. Grade 3 immune-related events included pneumonitis in 3 patients, and rash, aspartate aminotransferase elevation, diarrhea, and Guillain-Barre syndrome in 1 patient each. A subsequent Phase I/II trial combined BV/nivo with ipilumumab, a monoclonal antibody that inhibits cytotoxic T lymphocyte antigen-4 (CTLA-4) (12). Eighteen of 64 patients (28%) had relapsed after three or more prior lines of therapy, and 55% were refractory to their last therapy. Among 18 patients treated with BV/nivo, the ORR was 89% (CR 61%). One-year PFS was 70% for the cohort, and approximately 80% for the 22 patients who underwent autoSCT. Triplet therapy with ipilumumab/BV/nivo was associated with a CR rate of 73% and 1 year PFS of 80%, but with higher immune-mediated toxicity. In all treatment groups, grade 1-2 fatigue, elevated liver enzymes, rash, and diarrhea were common, and 2 patients died of pneumonitis. With median follow-up of 2.2 years, the median duration of response was not reached for BV/nivo and ipilumumab/BV/nivo (13).

The BV/nivo regimen has also been studied in a pediatric/young adult population, with similar high response rate and tolerability. In the CheckMate 744 study, patients with standard risk relapsed/refractory HL, age 5-30 years, were treated with 4 cycles of BV/nivo, followed by BV/bendamustine intensification for patients not in CR (14). Patients in CR received consolidation with autoSCT. Among 44 patients, CR rate was 59% after BV/nivo, and 25% of the study population received BV/bendamustine intensification. Ninety-five percent of patients underwent consolidation with autoSCT. With relatively short follow-up, 1-year PFS was 91%. The most common adverse events attributed to BV/nivo were nausea, hypersensitivity, and diarrhea. Immune-mediated adverse events were all grade 1-2. In the low risk arm of CheckMate 744, patients who achieved a CR after either BV/nivo alone or BV/nivo followed BV/bendamustine intensification were treated with involved site radiotherapy (ISRT), without consolidative autoSCT (15). Among 28 patients, 3-year event free survival was 87%, suggesting that a nivolumab-containing regimen may obviate the need for consolidative autoSCT in selected patients with relapsed HL.

Addition of pembrolizumab to multiagent chemotherapy regimens has produced high CR rates and durable remissions. In 42 adult patients relapsed after 1 or 2 lines of prior therapy (43% primary refractory, 32% relapsed within 1 year), pembrolizumab plus ICE (pembro-ICE) resulted in a CR rate of 87%, overall response rate of 97% and 2 year PFS after autoSCT of 87% (16). There was no increased toxicity noted over that expected with ICE. In a single institution Phase II study, pembrolizumab plus gemcitabine/vinorelbine/doxil (pembro-GVD) was studied as second-line therapy in 39 patients, 41% of whom were primary refractory and 38% relapsed within 1 year (17). Ninety-five percent of patients received consolidative autoSCT. CR rate was 92% and PFS was 100% at 13 months of follow-up. With follow-up extended to median 30 months, only one patient has relapsed (18). The study is ongoing with a second cohort who are treated with an extended course of pembrolizumab maintenance rather than consolidation with autoSCT. Pembro-GVD is associated with immune-related adverse events, including hyperthyroidism, transaminitis, and rash.

## PD-1 inhibitors in newly diagnosed HL

Standard treatment of early-stage HL with combination chemotherapy and radiotherapy is highly effective, but there remains room for improvement. Ten to fifteen percent of patients with unfavorable features like B-symptoms, elevated erythrocyte sedimentation rate, and bulky disease progress after frontline therapy, and omission of radiotherapy has consistently been associated with a decrement in PFS. Patients with advanced stage HL also could benefit from more effective frontline therapy. Multi-agent chemotherapy regimens like ABVD and BEACOPP provide long-term cure for the majority of patients with Stage III-IV HL, but 20-30% of patients do not have durable remissions with these regimens, and short- and long-term toxicities can be significant, particularly with BEACOPP.

The current standard of care for patients with advanced stage HL is BV-AVD, as established by the ECHELON-1 trial, which randomized adults with newly diagnosed Stage III-IV HL to ABVD versus BV-AVD. The study met its primary endpoint of modified 2-year PFS (82% v. 77%), although with more neutropenia and peripheral neuropathy in the BV-containing arm (19). With median follow-up of 73 months, OS was superior with BV-AVD (94% v. 89%) (20). Addition of BV to first line chemotherapy also improved PFS in a pediatric/adolescent population (21).

The remarkable activity of PD-1 inhibitors as single agents and in combination with chemotherapy in the relapsed/refractory setting has motivated clinical trials incorporating them into the frontline treatment of patients with HL (**Table 2**). The NIVAHL study assigned 109 patients with early-stage unfavorable risk HL to four cycles of either sequential or concurrent nivolumab and AVD (22). All patients received 30 Gy ISRT. The CR rate was greater than 90% in both arms, and 12-month PFS was 100% with concomitant therapy and 98% in the sequential arm. In the CheckMate 205 study, 51 patients with either unfavorable early stage or advanced stage HL received 4 cycles of nivolumab monotherapy followed by 6 cycles of nivo-AVD (23). Only 18% of patients achieved CR after nivolumab monotherapy, but overall and complete response rates at end of therapy were 93% and 74% in evaluable patients. With early follow-up, 9 month modified PFS was 92%. Of note, patients with higher expression of PD-L1 had a higher CR rate than those with the lowest quartile of PD-L1 expression.

**Table 2:** Phase II frontline trials incorporating PD1-inhibitors.

Regimen	Stage	N	CR	PFS	Ref
Nivolumab x 4→ N-AVD x 6	Early unfavorable and advanced	51	67%	92% at 9 mo.	(23)
Concurrent or sequential N-AVD x 4 + ISRT	Early unfavorable	109	92%	99% at 3 yr.	(42)
Pembrolizumab x 3→ AVD x 4-6	Early unfavorable and advanced	30	100%	100% at median 22.5 mo	(24)
Pembrolizumab-AVD x 2-6	All stages	30	90%	97% at 2 yr.	(27)
BV-nivolumab x 8	All stages	46*	48%	Median PFS 18 mo.	(29)

N-AVD, nivolumab/Adriamycin/vinblastine/dacarbazine; ISRT, involved site radiotherapy; BV, brentuximab vedotin.

*All ≥ 60 years or unfit for standard chemotherapy.

A phase II trial of sequential pembrolizumab and AVD in 30 patients with unfavorable early and advanced stage HL reported a 37% CR rate after 3 cycles of pembrolizumab and 100% at the end of AVD (24, 25), with no patient receiving radiotherapy. At median follow-up of 33 months, PFS and OS were 100%. In this study, there was not a correlation between response and PD-L1 or PD-L2 expression. An ongoing trial is investigating a risk-adapted approach of sequential pembrolizumab and chemotherapy in patients with early unfavorable or advanced stage cHL (26). Patients are treated with 3 cycles of pembrolizumab followed by 2 cycles of AVD. Patients who have a negative PET scan complete 2-4 additional cycles of AVD, and those with a positive PET scan proceed to 2-4 cycles of escalated BEACOPP. All patients receive consolidation with 4 cycles of pembrolizumab. Among the 146 enrolled patients, 29% had a negative PET after the initial course of pembrolizumab and 70% had a negative PET after the first 2 cycles of AVD.

One study reported to date has evaluated concurrent administration of pembrolizumab and AVD. The initial publication reported on 30 patients with advanced stage or unfavorable early-stage HL, with 73% completing 6 cycles of pembro-AVD (27). The regimen was associated with reversible grade 3 or 4 transaminase elevations in 10%, and 20% of patients missed one or more doses of pembrolizumab due to adverse events. Despite treatment interruptions in a substantial minority of patients, all patients responded, and CR rate was 90%. The results were updated after enrollment of an additional 20 patients (28). In the complete 50 patient cohort, only one patient has relapsed, and 2-year PFS is 98%. Of note, CR rate on interim PET scan after cycle 2 was 73% in early-stage patients and 58% in advanced stage patients, which is lower than expected after standard chemotherapy, although only one patient with positive interim PET scan has relapsed. Circulating tumor DNA may be more predictive than interim PET scan with this regimen, as more than 80% of patients had negative circulating DNA after cycle 2.

With the goal of minimizing toxicity while capitalizing on the activity of novel agents, the BV/nivo regimen was tested as first-line treatment for patients older than 60 years or with significant comorbidities (29). Forty-six patients were enrolled and 76% completed the protocol-specified 8 cycles of treatment. Unfortunately, the study did not meet the target for adequate response, with a CR rate of 48% and median PFS of 18 months. However, with a median follow-up of 21 months, more than half of patients with CR remain in remission, suggesting that a subset of older patients do benefit from this regimen.

The next iteration of frontline therapy in HL is the addition of both BV and nivolumab to standard chemotherapy. A regimen of BV/nivo plus adriamycin/dacarbazine has been evaluated in a cohort of 57 patients with early stage bulky or advanced stage HL, with a CR rate of 89%, overall response rate of 95%, and estimated 2 year PFS of 88% (30). Peripheral neuropathy occurred in 44%, but most events were grade 1-2. Immune-mediated adverse events included hypothyroidism (9%), pneumonitis (5%) and rash (5%). A second cohort of patients with non-bulky stage I-II disease is ongoing (31).

Although single-arm trials demonstrate the feasibility and efficacy of combining PD-1 inhibitors with standard chemotherapy, phase III trials are required to establish whether the regimens provide a clinically meaningful advantage over standard of care. The SWOG S1826 trial is poised to provide this evidence in advanced stage HL. This Phase III trial enrolled patients with newly diagnosed Stage III-IV HL, age 12 and older, including 25% under age 18 and 10% over age 61. Patients were randomized to 6 cycles of either nivo-AVD (n=489) or BV-AVD (n=487), with the primary endpoint of PFS. Prophylactic G-CSF was required with BV-AVD but was optional with nivo-AVD. The second interim analysis, with a median follow-up of 12.1 months, found one year PFS of 94% in the nivo-AVD arm, versus 86% in the BV-AVD arm (HR for PFS 0.48, p=0.0005) (32). Rates of febrile neutropenia, pneumonitis, transaminitis, and colitis were similar in both treatment arms. Hypo- and hyperthyroidism were more common in the nivolumab-containing arm, and peripheral sensory neuropathy was more common in the BV-containing arm. Fewer patients in the nivo-AVD arm received consolidative radiotherapy (0.4% v. 0.8%). The PFS benefit was present across all subgroups analyzed, including in the pediatric population (33). Patients older than 60 years particularly benefited from nivo-AVD, with higher PFS (93% v. 64%) and lower rates of infections, peripheral neuropathy, and GI symptoms (34).

## Discussion

In the fewer than 10 years since nivolumab and pembrolizumab were first reported to provide high response rates in patients with relapsed and refractory HL, the PD-1 inhibitors have steadily moved into earlier lines of therapy. Although no Phase III trials have compared traditional chemotherapy-based salvage regimens with nivolumab- or pembrolizumab-containing regimens, the results from Phase II trials support the use of PD-1 inhibitors for most transplant-eligible patients with relapsed HL. These trials have established that nivolumab and pembrolizumab can be safely incorporated into second line regimens, in combination with multi-agent chemotherapy or with brentuximab vedotin, providing high CR rates and excellent PFS after consolidative autoSCT. The choice of salvage regimen remains physician and patient dependent. BV/nivo has the longest follow-up, with 90% of patients who undergo subsequent autoSCT progression free at 3 years. It has a convenient outpatient schedule and is relatively well-tolerated. For patients who require chemotherapy to achieve CR before autoSCT, pembro-GVD has the highest reported CR rate (92%) with fewer than 5% of patients relapsing post-autoSCT. However, these results are based on a small single-institution study, and longer follow-up is needed. Pembro-ICE and nivo-ICE have the appeal of incorporating both a PD-1 inhibitor and a familiar potent chemotherapy combination.

The impressive efficacy of PD-1 inhibitors in second line has raised the question of whether some patients with relapsed HL can achieve long-term remission without autoSCT. A subset of patients with relapsed/refractory HL treated with BV/nivo do remain in remission for greater than 2 years (12, 29), and an ongoing study is investigating salvage pembro-GVD without consolidative autoSCT. To date, the strongest support for elimination of autoSCT comes from a very low-risk pediatric/young adult population treated with BV/nivo and ISRT (15). Longer follow-up and larger trials are needed to identify the patients with relapsed HL who are most likely to achieve long-term remission with a PD-1 containing salvage regimen alone, without consolidative autoSCT.

In newly diagnosed HL, Phase II trials of nivolumab or pembrolizumab added to a backbone of standard chemotherapy have shown promising efficacy, maintaining excellent response rates and PFS with the omission of bleomycin and radiation. The studies of sequential or concurrent nivo- or pembro-AVD have found that immune-mediated adverse events are generally low-grade and infrequent. The exception may be concurrent pembrolizumab and AVD, which is associated with higher rates of transaminitis. In the sequential trials, fewer than half of patients achieved a complete metabolic remission after single agent nivolumab or pembrolizumab, and spurious PET scan results may occur, so that patients often have durable remissions despite positive interim or end-of therapy PET.

Based on the early results from the Phase III trial of BV-AVD versus nivo-AVD, it is likely that PD-1 inhibitors will become standard of care in first line treatment of advanced stage HL in the near future. As reported in the S1826 study, nivo-AVD is a more tolerable front-line regimen, especially for patients older than 60 years, and its early PFS is statistically superior to the current standard, BV-AVD. Randomized trials will be needed to establish the relative efficacy of PD-1 containing regimens in frontline treatment of early-stage HL, but they hold out the appealing possibility of eliminating the need for consolidative radiation. The role of PD-1 inhibition in the frontline therapy of early stage HL will be further elucidated by the ongoing phase III AHOD2131 trial, in which patients are randomized to BV/nivo versus standard of care after 2 cycles of ABVD (35).

Biomarkers could provide critical guidance in the choice of regimen and early assessment of response, in both the frontline and relapsed settings. Future studies should investigate whether levels of PD-L1 and CD30 expression predict response to regimens incorporating PD-1 inhibitors or BV. Biomarkers like circulating cell-free DNA, TARC (Thymus and Activation Regulated Chemokine), and total metabolic tumor volume have demonstrated prognostic or predictive value in prior studies, and they should be further explored in randomized trials of PD-1 inhibitor regimens.

As more patients are treated with PD-1 inhibitors in the frontline, the management of relapsed/refractory patients will need to adapt. Salvage regimens that include checkpoint inhibitors have not been studied in patients previously treated with nivolumab or pembrolizumab, although patients who were not refractory to frontline use of these agents may respond to re-treatment. Identification of patients with relapsed HL who can be cured without an autoSCT remains a worthy goal, but one that will have to adjust to the patient’s prior therapy and duration of response. In particular, patients who have primary refractory disease or early relapse after PD-1 containing frontline therapy are likely to require autoSCT to achieve durable response in second line therapy. Future efforts to optimize the integration of PD-1 inhibitors in frontline and salvage regimens have the potential to minimize exposure to chemotherapy and radiation and to reduce the number of patients who require autoSCT.
